# Assessing White Matter Pathology in Early-Stage Parkinson Disease Using Diffusion MRI: A Systematic Review

**DOI:** 10.3389/fneur.2020.00314

**Published:** 2020-05-14

**Authors:** Maurizio Bergamino, Elizabeth G. Keeling, Virendra R. Mishra, Ashley M. Stokes, Ryan R. Walsh

**Affiliations:** ^1^Division of Neuroimaging Research, Barrow Neurological Institute, Phoenix, AZ, United States; ^2^School of Life Sciences, Arizona State University, Tempe, AZ, United States; ^3^Imaging Research, Cleveland Clinic Lou Ruvo Center for Brain Health, Las Vegas, NV, United States; ^4^Muhammad Ali Parkinson Center, Barrow Neurological Institute, Phoenix, AZ, United States

**Keywords:** MRI diffusion, early-stage Parkinson disease, diffusion tensor imaging, diffusion kurtosis imaging, q-space diffeomorphic reconstruction, fractional anisotropy, substantia nigra

## Abstract

Structural brain white matter (WM) changes such as axonal caliber, density, myelination, and orientation, along with WM-dependent structural connectivity, may be impacted early in Parkinson disease (PD). Diffusion magnetic resonance imaging (dMRI) has been used extensively to understand such pathological WM changes, and the focus of this systematic review is to understand both the methods utilized and their corresponding results in the context of early-stage PD. Diffusion tensor imaging (DTI) is the most commonly utilized method to probe WM pathological changes. Previous studies have suggested that DTI metrics are sensitive in capturing early disease-associated WM changes in preclinical symptomatic regions such as olfactory regions and the substantia nigra, which is considered to be a hallmark of PD pathology and progression. Postprocessing analytic approaches include region of interest–based analysis, voxel-based analysis, skeletonized approaches, and connectome analysis, each with unique advantages and challenges. While DTI has been used extensively to study WM disorganization in early-stage PD, it has several limitations, including an inability to resolve multiple fiber orientations within each voxel and sensitivity to partial volume effects. Given the subtle changes associated with early-stage PD, these limitations result in inaccuracies that severely impact the reliability of DTI-based metrics as potential biomarkers. To overcome these limitations, advanced dMRI acquisition and analysis methods have been employed, including diffusion kurtosis imaging and q-space diffeomorphic reconstruction. The combination of improved acquisition and analysis in DTI may yield novel and accurate information related to WM-associated changes in early-stage PD. In the current article, we present a systematic and critical review of dMRI studies in early-stage PD, with a focus on recent advances in DTI methodology. Yielding novel metrics, these advanced methods have been shown to detect diffuse WM changes in early-stage PD. These findings support the notion of early axonal damage in PD and suggest that WM pathology may go unrecognized until symptoms appear. Finally, the advantages and disadvantages of different dMRI techniques, analysis methods, and software employed are discussed in the context of PD-related pathology.

## Introduction

Parkinson disease (PD) is a chronic progressive neurodegenerative disease that affects more than 10 million people worldwide (1–3). Parkinson disease pathology is characterized by Lewy body aggregates and neurites (4, 5), which play a causative role in degeneration of dopaminergic neurons in the substantia nigra (SN); motor symptoms associated with PD have been primarily attributed to this process (6). By the time PD becomes symptomatic, an estimated 60% of dopaminergic neurons have degenerated, representing a moderate to severe state of disease (7). Biomarkers, including fluid and imaging based, may play a critical role in understanding the natural history of progression of PD and enable appropriate therapeutic intervention, thereby optimizing preservation of neural health (8). However, to date, no definitive biomarker exists for this purpose, despite the clear need for one.

Imaging-based biomarkers for PD can yield insight into atrophy, microstructural changes, neuronal activity, and vascular hemodynamics. Magnetic resonance imaging (MRI) biomarkers, both structural and functional, are increasingly used in PD for more comprehensive evaluation of neuropathology. Subtle brain atrophy has been demonstrated in PD using voxel-based morphometry (VBM), which is an automated analysis approach to characterize volumetric brain changes from three-dimensional structural imaging (9–11). Altered patterns of neuronal activation, measured via functional MRI (fMRI), have been observed in multiple regions of the cortex in PD using a range of motoric tasks (12–14). Resting-state fMRI is measured in the absence of tasks and can identify abnormalities in spontaneous neuronal activity (15). In particular, this approach has revealed changes in the corticosubcortical functional connectivity in PD compared with healthy controls (HCs) (16).

Diffusion MRI (dMRI) comprises a set of complementary techniques to non-invasively probe microstructural characteristics via diffusivity of water molecules in the brain. As water predominantly diffuses along axons, dMRI can be used to probe white matter (WM) changes such as axonal caliber, density, myelination, and orientation. Diffusion tensor imaging (DTI), the most common dMRI model, can provide measures such as fractional anisotropy (FA), mean diffusivity (MD), axial diffusivity (AxD), and radial diffusivity (RD) (17) that are sensitive to subtle WM microstructural organization. Since the inception of DTI in the mid-1990s (18), significant improvements have been made in both acquisition and analysis methods. These advances include the implementation of multishell and high-angular-resolution dMRI data, such as those with more diffusion-encoding gradient directions, resulting in the development of advanced algorithms to improve dMRI postprocessing and overcome some of the known limitations of DTI. The availability of standard software has further enabled quantitative analysis of DTI and DTI-related metrics including advanced dMRI models. At present, DTI represents one of the most widely used neuroimaging methods, in both preclinical animal and human studies, for its versatility and specificity to WM microstructure (19). We encourage interested readers to refer to References (9, 20–23) for a more in-depth understanding of DTI.

White matter changes in PD vs. controls (healthy subjects; HCs) have previously been evaluated by DTI using various metrics, acquisitions, analyses, and software tools (24). More specifically, a recent meta-analysis encompassing wide disease duration DTI studies in PD found that both FA and MD were able to distinguish between PD and HC, with regional DTI changes observed in the SN, corpus callosum, cingulate, and temporal cortices. Considering the growing interest in defining the early phases of PD through the use of neuroimaging biomarkers, the focus of this systematic review is the current state of dMRI-based biomarkers for understanding early-stage PD, with an emphasis on the technical methodologies employed for dMRI. More specifically, the basic theoretical background for standard DTI will be provided, along with three more advanced methods that go beyond standard DTI methods [diffusion kurtosis imaging (DKI), neurite orientation dispersion and density imaging (NODDI), and Q-space diffeomorphic reconstruction (QSDR)]. The relevant results in early-stage PD will be discussed for each of these methods. Additionally, the advantages and disadvantages of different dMRI acquisition techniques, analysis methods, and software employed will be discussed in the context of early-stage PD-related pathology. The commonalities and incongruences in findings in early-stage PD will be contextualized between the various dMRI methods. Finally, given the current state of understanding of early-stage PD and known capabilities of dMRI approaches, the outlook for future opportunities in dMRI to improve pathophysiological characterization of early-stage PD will be discussed.

## Methods

**Figure 1:**
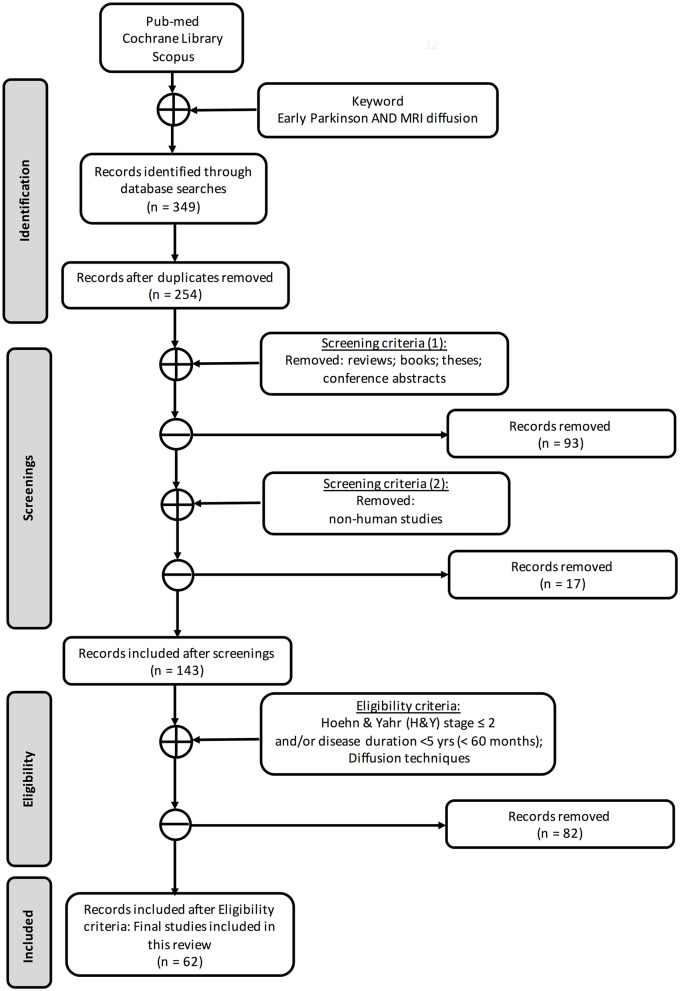
Search strategy based on PRISMA flow diagram.

Following Preferred Reporting Items for Systematic Reviews and Meta-Analyses (PRISMA) guidelines (25), we searched three databases (Figure 1) for works that included at least one group of early PD [defined as subjects with Hoehn & Yahr (H&Y) stage ≤2 and/or disease duration <5 years] and where one or more dMRI methods were used (see PRISMA diagram in Figure 1). To identify the articles for this systematic review, we searched for publications on PubMed, Cochrane Library, and Scopus databases by using the keywords “early Parkinson,” “MRI diffusion,” and “DTI” without any temporal restriction. In total, we found 349 records. After excluding duplicates (*n* = 254), screening and eligibility further reduced the number to 62 articles retained for this review.

**Figure 2:**
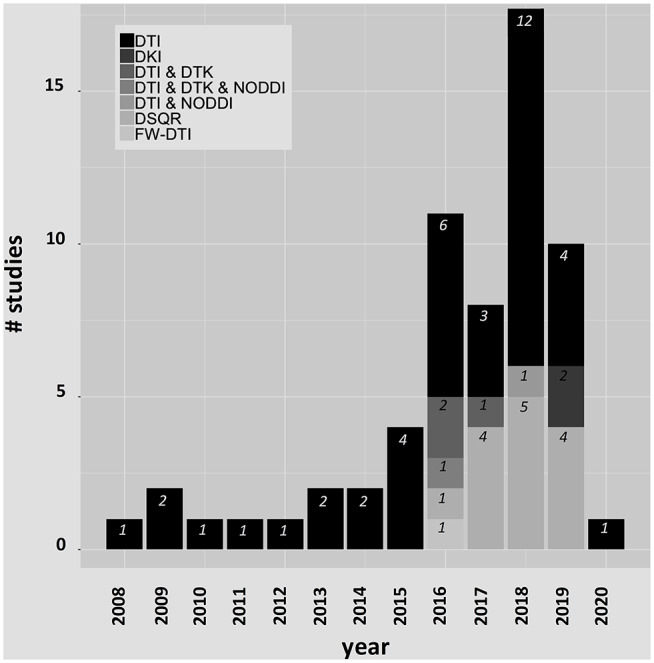
Histograms for the publication years and dMRI techniques included in this review.

While most studies used site-specific data, several studies leveraged the open-access availability of data from the Parkinson's Progression Markers Initiative (PPMI) (http://www.ppmi-info.org). Parkinson's Progression Markers Initiative is a multicenter international database of *de novo* individuals with early idiopathic PD that includes clinical, imaging, and biological data. Studies using PPMI and other shared datasets will be denoted in the text. Histograms are shown in Figure 2 for the publication years and dMRI techniques used in each article included in this review. The complete list of the PD studies that are included in this review is reported in Table 1.

**Table 1:** Summary of diffusion studies in early-stage PD.

**References**	**Diffusion method**	**Analysis method (for diffusion)**	**Software**	**PD subjects**	**# Diffusion directions/# *b* value(s)**
Vriend et al. (26)	DTI	Connectivity	FSL, BCT	23 early-stage PD (H&Y 1–3)	30/2
Arrigo et al. (27)	DTI	Connectivity	SPM8, FSL, MRtrix, Camino	20 newly diagnosed PD (H&Y = 1)	61/2
Tinaz et al. (28)	DTI	Connectivity	BCT, FATCAT, 3dTrackID, TORTOISE, AFNI	20 non-demented PD patients (H&Y = 2; disease duration (year) = 7.1)	70/—
Peña-Nogales et al. (29)	DTI	Connectivity	FSL–MRtrix	PPMI subjects	64/2
Nigro et al. (30)	DTI	Connectivity	PANDA (Matlab) –BCT–FSL	H&Y = 1.5 and disease duration = 19.28 months	27/2
Tessa et al. (31)	DTI	Histogram	FSL	27 patients with *de novo* drug-naive PD [tremor-dominant type (n = 13), akinetic-rigid type (n = 11), and mixed type (n = 3)]–H&Y = 1.2 and 1	6/2
Knossalla et al. (32)	DTI	ROI	—	10 early-stage PD (H&Y = 1–2)	20/2
Joshi et al. (33)	DTI	ROI	FSL	24 early-stage PD (disease duration average 2.94 years, SD 2.93 years; nine HY1 patients, 13 HY2, two unknown)	55/—
Wang et al. (34)	DTI	ROI	Siemens Syngo MR Neuro 3D, SPM8, FSL	27 early-stage PD (H&Y = 1–2; duration of disease = 1.7 Y)	60/2
Aquino et al. (35)	DTI	ROI	FSL	22 early-stage PD (Duration of disease = 4.0 years) and 20 late PD	64/—
Vaillancourt et al. (36)	DTI	ROI	AFNI	14 with early stage PD (H&Y = 1–2; duration of disease = <34 months)	27/2
Gattellaro et al. (37)	DTI	ROI	ImageJ (for ROI)	10 PD without dementia (H&Y = 1–2)	12/2
Du et al. (38)	DTI	ROI	DTIPrep, Matlab	15 early-stage PD (disease duration ≤ 1 years), 14 midstage PD (duration 2–5 years), and 11 late-stage (duration >5 years)	42/2
Loane et al. (39)	DTI	ROI	ExploreDTI	18 early stage PD (treated) [avg (SD) disease duration (years): 3.9 (2.2); no H&Y provided]	64/2
Schuff et al. (40)	DTI	ROI	Processed from PPMI	PPMI subjects	64/2
Pelizzari et al. (41)	DTI	ROI	FSL, ANTs	26 PD (H&Y = 1–1.6; duration of disease = 3.0 years)	64/2
Guan et al. (42)	DKI	ROI	GE adw 4.6	The PD divided into an advanced-stage PD group and an early-stage PD group	15/3
Liu et al. (43)	DTI	ROI	Probably Scanner software	early diagnosis of Parkinson disease	25/2
Mangia et al. (44)	DTI	ROI	FSL	Nine early-diagnosed PD	93/2
Klein et al. (45)	DTI	ROI	—	20 early-stage PD patients (disease duration 1.9 ± 0.97 years, H&Y 1–2)	60/2
Planetta et al. (46)	DTI	ROI, tractography	DTI Studio, AFNI,	20 with early stage PD (Duration of disease = 12 months)	27/2
Wei et al. (47)	DTI	ROI, tractography	GE adw 4.5	21 early (H&Y <2) and 22 mid–late PD (H&Y ≥2)	25/2
Li et al. (48)	DTI	TBSS	FSL	31 early-stage PD (H&Y = 1–2)	32/2
Rolheiser et al. (49)	DTI	TBSS	FSL	14 early stage PD (H&Y = 1–2; duration of disease <72 months)	31/2
Ibarretxe-Bilbao et al. (50)	DTI	TBSS	FSL	24 early-stage PD (H&Y = 1–2)	30/2
Minett et al. (51)	DTI	TBSS	FSL	120 early stage PD [27 with mild cognitive impairment (H&Y = 2.3) and 93 with normal cognition (H&Y = 1.9)]; duration of disease 5.6–6.4 months (longitudinal study)	64/2
Duncan et al. (52)	DTI	TBSS	FSL	125 non-demented PD (H&Y = 2.0; average duration of disease = 6.15)	64/2
Lacey et al. (53)	DTI	TBSS	FSL	PPMI subjects	64/2
Pozorski et al. (54)	DTI	TBSS	FSL–DTIprep–DTI_TK	H&Y stage at baseline 16 subjects with <2 and 13 subjects (≥2); mean disease duration (years) 3.7 (3.2)	40/2
Rektor et al. (55)	DTI	TBSS	FSL	H&Y stage 1–1.5 and disease duration up to 5 years	60/2
Pelizzari et al. (56)	DTI	TBSS	FSL	12 PD [median H&Y (IQR) = 1.5 (1.1–2)]	64/2
Chen et al. (57)	DTI	TBSS (only for normalization procedure), ROI	FSL	30 early stage PD (H&Y = 1.74; average duration of disease = 62)	25/2
Mishra et al. (58)	DTI	TBSS (skeleton), ROI, VBA	FSL, DTI-TK	PPMI subjects	64/2
Gou et al. (59)	DTI	TBSS, Connectivity	FSL, SPM12, PANDA, FACT	PPMI subjects	64/2
Meijer et al. (60)	DTI	TBSS, ROI	FSL	49 early stage PD [19 atypical parkinsonism (H&Y = 2.4) and 30 PD (H&Y = 1.7)], disease = 21.6–28.4 months. Longitudinal study	30/2
Guimarães et al. (61)	DTI	TBSS, ROI, tractography	FSL, Explore DTI, SPM8	early-stage PD, moderate PD, and severe PD	32/2
Prange et al. (62)	DTI	TBSS, VBM	FSL	14 apathetic and 13 non-apathetic patients with *de novo* PD	24/2
Ford et al. (63)	DTI	TBSS, VBM	FSL, SPM8 (for VBM)	124 early-stage PD	64/2
Zhang et al. (64)	DTI	Tractography	SPM8, TrackVis	PPMI subjects	64/2
Lorio et al. (65)	DTI	VBA	SPM12, FSL	PPMI subjects	64/2
Taylor et al. (66)	DTI	VBA	TEEM tool (from PPMI)	PPMI subjects	64/2
Planetta et al. (67)	DTI (free water)	ROI	FSL–Matlab for free water	34 patients with early stage PD	64/2
Rahmani et al. (68)	QSDR	Connectivity	Explore DTI–DSI Studio	PPMI subjects	64/2
Ghazi Sherbaf et al. (69)	QSDR	Connectivity	Explore DTI–DSI Studio	PPMI subjects	64/2
Ansari et al. (70)	QSDR	Connectivity	Explore DTI–DSI Studio	PPMI subjects	64/2
Ansari et al. (71)	QSDR	Connectivity	DSI Studio	PPMI subjects	64/2
Ghazi Sherbaf et al. (72)	QSDR	Connectivity	DSI Studio–Explore DTI	PPMI subjects	64/2
Wen et al. (73)	QSDR	Connectivity	FSL, DSI Studio	20 prodromal phase of PD; 106 PD;	64/2
Haghshomar et al. (74)	QSDR	Connectivity	ExploreDTI, DSI-Studio	PPMI subjects	64/2
Ashraf-Ganjouei et al. (75)	QSDR	Connectivity	DSI Studio	PPMI subjects	64/2
Sanjari Moghaddam et al. (76)	QSDR	Connectivity	DSI Studio–Explore DTI	PPMI subjects	64/2
Sobhani et al. (77)	QSDR	Connectivity	DSI Studio	PPMI subjects	64/2
Ghazi Sherbaf et al. (78)	QSDR	ROI	DSI Studio, ExploreDTI	PPMI subjects	64/2
Wen et al. (79)	QSDR	TBSS–Connectivity	DSI Studio–FSL	PPMI subjects	64/2
Wen et al. (80)	QSDR	TBSS, Connectivity	FSL, DSI Studio, BCT,	PPMI subjects	64/2
Wen et al. (81)	QSDR	TBSS, Connectivity	FSL, DSI Studio, BCT	PPMI subjects	64/2
Zhang et al. (82)	DKI	ROI	GE adw 4.5	Initial H&Y staging 1.58 and 1.65; H&Y staging after 2 years 2.08 and 1.84	25/3
Zhang et al. (83)	DKI	ROI	GE adw 4.5	72 early-stage PD (H&Y = 1.67; duration of disease = 13.50 months)	25/2
Zhang et al. (84)	DKI	ROI	GE adw 4.5	28 PD with Striatal silent lacunar infarction PD (H&Y = 1.68 -> 2.39(FU); duration of disease = 14.21 months); 32 PD et al. [H&Y = 1.63 to >1.91 (FU); duration of disease = 14.68 months]	25/2
Zhang et al. (85)	DKI	ROI	GE adw 4.5	72 with early stage PD divided in control and striatal silent lacunar infarction (H&Y = 1.63 and 1.71; duration of disease <14 months)	25/2
Surova et al. (86)	DTI–DKI–NODDI	ROI, tractography	FSL, in-house developed software (for DKI)	105 patients with PD et al. [H&Y = 2; disease duration (years) = 5]	94/4
Andica et al. (87)	DTI–NODDI	Tractography	NODDI Matlab Toolbox5, FSL, AMICO, TrackVis	29 PD (H&Y = 1.97; average duration of disease = 6.24 years)	32/2

—*, no information available. In the last column, the B0 image acquisition is included in the number of b values*.

## Theory and Results

### Diffusion Tensor Imaging: Theory, Acquisition, and Analysis

Diffusion tensor imaging relies on motion-sensitizing gradients to probe the displacement of water molecules, which is often simplified using a Gaussian distribution model. The diffusion-weighted signal intensity for this distribution can be described by the following equation:

(1) $$\begin{array}{l} S=S_{0}exp\left[-bD\right] \end{array}$$

where *S*_0_ is the signal intensity without the diffusion gradient, *D* is the diffusion coefficient, and *b* is the diffusion-weighting factor, where the *b* factor is largely dependent on the gradient waveform. The diffusion can thus be represented by the following equation:

(2) $$\begin{array}{l} ln\left[\frac{S}{S_{0}}\right]=-b{\bar{g}}^{T}\bar{\;D}\;\bar{g} \end{array}$$

where $\bar{g}$ is a three-element column vector representing a gradient direction, ${\bar{g}}^{T}$ is the transpose of $\bar{g}$, and $\bar{\;D}$ is the apparent diffusion tensor (3 × 3 symmetric matrix).

Diffusion tensor imaging provides a direct relationship between the chosen experimental parameters, such as *b* and $\bar{g}$, the MR measurements (*S* and *S*_0_), and the parameters of the diffusion tensor model $\bar{D}$.

Eigendecomposition of the diffusion matrix yields a symmetric diffusion tensor:

(3) $$\begin{array}{l} \bar{D}=\left[\begin{array}{c} \epsilon_{1}\;\epsilon_{2}\;\epsilon_{3} \end{array}\right]\cdot \left[\begin{array}{c} \lambda_{1}\;0\;0\\ 0\;\lambda_{2}\;0\\ 0\;0\;\lambda_{3} \end{array}\right]\cdot \left[\begin{array}{c} \epsilon_{1}\\ \epsilon_{2}\\ \epsilon_{3} \end{array}\right] \end{array}$$

where λ_1_, λ_2_, and λ_3_ are the eigenvalues (with λ_1_ ≥ λ_2_ ≥ λ_3_) and ϵ_1_, ϵ_2_, and ϵ_3_ are the eigenvectors of $\bar{D}$. The diffusion tensor is completely characterized by these eigenvalues, which describe the length of the three axes of the diffusion ellipsoid, and their corresponding eigenvectors, which describe the orientation of these axes in space. As the eigenvectors provide information about the direction of water diffusion within a voxel, they form the basis of brain fiber tracking (88, 89). The geometric shape associated with the diffusion tensor is assumed to be a three-dimensional ellipsoid with the length of the three orthogonal principal axes proportional to the ordered tensor eigenvalues.

The first eigenvalue (λ_1_) represents water diffusivity along the principal axis and is termed axial (or longitudinal or parallel) diffusivity (λ_∥_ or AxD). The radial (or transverse or perpendicular) diffusivity (λ_⊥_ or RD) represents water diffusion perpendicular to the principal direction and is given by the average of the remaining eigenvalues ((λ_2_ + λ_3_)/2). Axial diffusivity has been associated with axonal damage, whereas RD may be associated with myelin integrity, axonal diameter and density, and fiber coherence (90, 91). Mean diffusivity is a rotationally invariant metric, obtained from a simple average of the diffusion eigenvectors ((λ_1_ + λ_2_ + λ_3_)/3), which describes the overall size of the tensor.

Fractional anisotropy quantifies the degree of anisotropy of the diffusion tensor and is the most common DTI-related index. Fractional anisotropy ranges from 0 to 1, where 0 represents isotropic diffusion and 1 represents completely anisotropic diffusion. Fractional anisotropy can be calculated in each voxel using the following equation:

(4) $$\begin{array}{l} FA=\sqrt{\frac{1}{2}}\frac{\sqrt{{\left(\lambda_{1}-\lambda_{2}\right)}^{2}+{\left(\lambda_{2}-\lambda_{3}\right)}^{2}+{\left(\lambda_{3}-\lambda_{1}\right)}^{2}}}{\sqrt{\lambda_{1}^{2}+\lambda_{2}^{2}+\lambda_{3}^{2}}}\;\\ \; \end{array}$$

**Figure 3:**
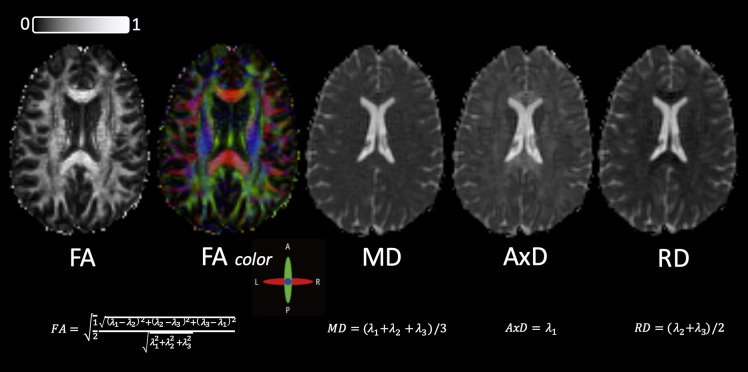
Standard DTI maps, from a healthy volunteer, created by DSI Studio with a DTI diffusion scheme with a total of 67 diffusion sampling directions. The *b* value was 1,000 s/mm^2^. The in-plane resolution was 2 mm, and the slice thickness was 2 mm. FA, fractional anisotropy; MD, mean diffusivity; AxD, axial diffusivity; RD, radial diffusivity. The range of FA in between 0 (isotropic diffusion) and 1 (anisotropic diffusion).

While this index has often been interpreted as a quantitative biomarker of WM disorganization, equating FA with WM disorganization is not strictly accurate, given that FA cannot disentangle the individual microscopic contributions, such as different WM fiber populations and/or cerebrospinal fluid (CSF) contamination (92, 93). Fractional anisotropy has also been equated as a marker of demyelination (94), which is also not accurate because the regional anisotropy may also reflect altered axonal diameter, packing density, or membrane permeability (95). Despite these limitations, DTI-based FA has often been utilized as a neuroimaging biomarker because of its robustness to noise (96, 97). Example maps for FA (gray-scale and colorized), MD, AxD, and RD are shown in Figure 3 in an HC.

**Figure 4:**
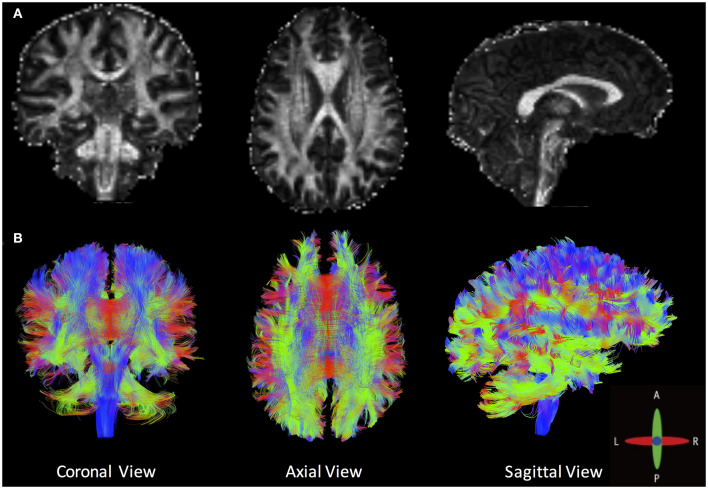
**(A)** Fractional anisotropy maps and **(B)** an example of whole-brain deterministic tractography created by DSI Studio in a healthy volunteer (angular threshold: 60 degrees; step size: 1 mm; anisotropy threshold: 0.20. Tracks with length shorter than 50 or longer than 300 mm were discarded. A total of 500,000 seeds were placed inside the whole brain).

Tractography goes beyond the conventional quantitative voxel-wise metrics to generate a three-dimensional representation of WM fiber bundles. Figure 4 shows an example tractography (B), with FA shown in (A), in an HC. Tractography consists of a multistep procedure to reconstruct WM fiber bundles inside the brain, namely, seeding, propagation, and termination. Many options, and best practices, for each of these steps can be found in the literature (23, 98), and software exists to perform these functions at varying levels. Some of the known limitations of tractography are related to acquisition (minimum number of DTI directions) and WM architecture (crossing, kissing, and diverging fibers). Other limitations include possible inaccuracies of tractography due to the presence of neuropathological changes (99).

**Figure 5:**
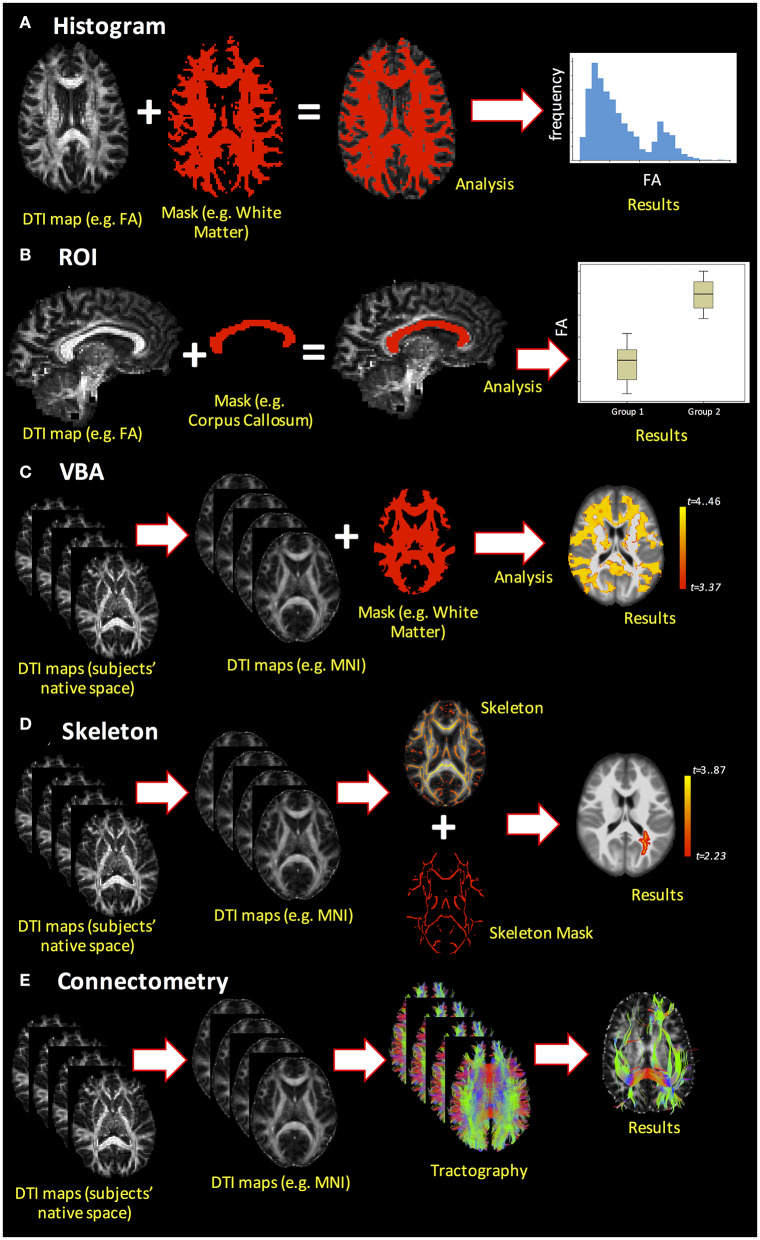
Example of the different dMRI analysis methods. **(A)** Histogram analysis; **(B)** ROI analysis; **(C)** voxel-based analysis (VBA); **(D)** skeletonized analysis (e.g., TBSS pipeline); **(E)** connectome analysis.

Upon generation of DTI-based metrics, subsequent quantitative analyses can be performed using histogram analysis, region of interest (ROI) analysis, voxel-based analysis (VBA), or skeletonized analysis [also called tract-based spatial statistics (TBSS)]. Additionally, DTI enables measurement of the macroscopic orientation of WM tissue and analysis of structural connectivity through tractography algorithms. These different analysis methods are demonstrated in Figure 5 and are discussed in greater detail below.

One of the simplest analyses is whole-brain histogram analysis (Figure 5A). Beyond mean and median, histograms permit extraction of parameters such as histogram peak height and location that can be compared across subjects or correlated with other variables. A major advantage of whole-brain histogram analysis is that no *a priori* choice of region is required; however, one ensuing drawback is that the results may be affected by CSF contamination (92). Region-of-interest analysis (Figure 5B) is a commonly used method to analyze DTI-derived indices, where ROIs can be obtained from automated segmentation (e.g., FreeSurfer; https://surfer.nmr.mgh.harvard.edu/) or by manual delineation. Although ROIs can be drawn directly on the DTI-derived indices, their placement may be difficult due to low-resolution images and the intensity of DTI-derived maps (e.g., FA) may spuriously influence the ROI boundaries, thereby introducing bias into the analysis. Alternatively, ROIs may be drawn on anatomical T1- or T2-weighted images, which necessitates reliable co-registration with dMRI data. One drawback to ROI analysis is that it requires either an *a priori* hypothesis regarding where WM differences are expected to be present in a pathology or normal development as the inclusion of many ROIs increases the number of statistical tests and requires correction for multiple comparisons.

Moving beyond ROI analysis, VBA is a fully automated approach that allows for the investigation of microstructural organization in each voxel inside the whole brain (Figure 5C). It involves the spatial normalization of high- and low-resolution images from the subjects' native space to stereotactic space, and thus, reliable co-registration is crucial. Additionally, smoothing of the data during analysis can increase the impact of partial volume effects (PVE), as the voxels may combine both WM and gray matter (GM) in such a way that the results are less robust and less specific to a specific component. As statistical analysis is performed in each voxel, there is also an increased risk of false-positive findings, such that multiple comparison corrections are compulsory. Finally, pathologies and lesions can strongly affect VBA results. Alternatively, TBSS (or skeletonized analysis, Figure 5D) can alleviate the alignment and smoothing challenges associated with VBA (100). Tract-based spatial statistics is a popular pipeline used to coregister sets of DTI maps for performing voxel-wise comparisons on skeletonized WM tracts. As a result, it precludes the study of whole-brain WM tracts, focusing instead on the components of WM tracts common across all subjects (hence the name skeletonized). In recent years, several studies have questioned the reliability and interpretability of TBSS (101), and improvements over the original TBSS pipeline have been suggested (102).

The final analysis method covered to this review is the connectome analysis (Figure 5E), which pertains to whether there are global changes in structural connectivity patterns at the end of WM pathways (103). Advantages of this method include that it is automated and identifies the global shift in WM connectivity pattern between the groups. Network-based measures derived through this connectome analysis can then be utilized to not only understand whether there is a global shift in WM-derived structural connectivity due to pathology but also to understand its correlations with clinical and pathological presentations.

### DTI in Early-Stage PD

Given the known spatiotemporal progression of changes associated with PD, ROI analysis has been used widely in early-stage PD. Moreover, given the recognized involvement of the SN early in pathophysiological progression of PD, many DTI studies have focused on ROI analysis of the SN. For example, reduced FA of SN in subjects with early-stage PD compared with controls was observed by Vaillancourt et al. (36), whereas Liu et al. (43) similarly found that FAs in the rostral, middle, and caudal areas of the SN were decreased significantly in subjects with early-stage PD compared with controls. Mangia et al. (44) explored the SN and other brain locations from a multimodal MRI approach and reported neuronal degeneration of the SN in the early-PD group. However, not all studies have reported similar results. For instance, Pellizari et al. (41) observed no differences in FA in the SN between early-stage PD and HC, although differences in AxD were reported. Similarly, Joshi et al. (33) compared all DTI-derived metrics in 24 early-stage PD subjects and found increased MD in the SN. Finally, in a longitudinal study over almost 2 years, Loane et al. (39) found no significant differences in DTI metrics at baseline, but significant differences in nigral FA (decrease in PD) and MD (increase in PD) metrics were observed at the follow-up time point. As a result, the authors hypothesized that diffusion metrics in the SN may be sensitive measures of disease progression.

Parkinson disease is often associated with olfactory dysfunction, and impaired sense of smell is one of the earliest clinical symptoms of PD, preceding even the classic motor signs (104). Using ROI analysis, significant group differences in FA and MD have been observed in the anterior olfactory structures (33). These results were confirmed by Rolheiser et al. (49), who leveraged TBSS to reveal significant group differences between PD and controls in the anterior olfactory region, as well as the SN. Furthermore, reduced FA in WM associated with the central olfactory system was observed using TBSS in early-stage PD patients and was associated with a reduced ability to smell (50).

Parkinson disease–associated pathological changes are not isolated to the SN and olfactory regions. In fact, alterations have been observed in other WM areas, including the genu of the corpus callosum, superior longitudinal fasciculus, putamen, external capsule, midbrain, superior cerebellum, and superior cerebellar peduncles (37, 60). Using both ROI and tractography methods, Planetta et al. (46) found that FA values were significantly reduced in PD in fibers projecting from the anterior nucleus, ventral anterior nucleus, and dorsomedial nucleus. In addition, reduced FA values approached significance in the ventral lateral nucleus of patients with PD. Similarly, regions that have shown reduced FA using TBSS include the bilateral anterior corona radiata, upper corona radiata, posterior thalamic radiation, optic radiation, fornix, and corpus callosum, among others (48, 56). In a study of 125 early-stage PD and 50 HC individuals, increased MD in PD subjects was observed in several WM locations, suggesting early axonal damage (52). On the other hand, some studies have reported no significant differences between early-stage PD and controls (35, 39), whereas higher FA values in several WM locations have also been reported (57).

Given the evidence of widespread WM changes, whole-brain metrics may be of interest. Tessa et al. (31) previously used whole-brain histogram analysis to compare FA metrics between controls and *de novo* drug-naive PD (*n* = 27), the latter of which was divided in tremor-dominant, akinetic-rigid, and mixed types. Increased FA was observed in patients with PD, which was more pronounced in patients with the akinetic-rigid subtype. Considering the use of whole-brain analysis, these results support the hypothesis that widespread neuropathology exists at the time of clinical onset, possibly driven by the inclusion of GM in whole-brain analysis. Connectome analysis has also shown significant differences in the WM-derived structural connectome associated with PD (29, 30, 105). More specifically, lower global efficiency and global clustering coefficient have been observed in PD compared with HCs (26). Arrigo et al. (27) found significant alterations in optic radiation connectivity distribution, a significant increase in optic radiation MD, and a significant reduction in WM concentration in early-stage PD. Finally, Tinaz et al. (28) investigated the structural and functional organization in PD subjects, finding reduced WM connectivity in frontoparietal–striatal nodes compared to controls, but no change in modular organization of the WM tracts. A recent study by Mishra et al. (105) reported that there is an early-stage PD-specific WM-derived connectome comprising pathophysiologically relevant regions and that the overall connectivity in early-stage PD in these regions is significantly higher than that compared to HC. Additionally, PD groups have shown reduction in functional local network metrics in many nodes distributed across the connectome (28).

Several studies have sought to correlate DTI findings with clinical metrics, including functional and cognitive measures, both cross-sectionally and longitudinally. The cross-sectional results have been mixed with both ROI analysis and TBSS, with some studies showing no correlations between DTI-derived metrics and clinical measures (38, 48) and other studies reporting significant correlations, particularly in the caudate nucleus (34, 41). Minett et al. (51) found that at baseline patients with early-stage PD had significantly higher MD relative to HC, and in patients with PD and mild cognitive impairment, higher MD was significantly correlated with lower attention and executive function scores. In this longitudinal study, DTI-derived WM microstructural changes were assessed as potential prognostic biomarkers of worsening motor features or cognitive decline in patients with PD. At follow-up, frontal MD increased significantly when comparing patients with PD and mild cognitive impairment with HC. In another longitudinal study over 18 months, patients with early-stage PD with unilateral disease were shown to have higher rates of microstructural changes compared to patients with later-stage PD with bilateral disease, suggesting that substantial microstructural changes occur during the early stages of disease (54). Gray matter changes have also been implicated in PD-associated cognitive impairment, where reduced cortical microstructural integrity was associated with reduced cognitive performance in early-stage PD patients (45).

While many of the aforementioned studies have used subject sample sizes on the order of 20 to 30, several studies have leveraged the PPMI database that features larger patient enrollment and longitudinal data, along with clinical metrics. Using ROI analysis, Schuff et al. (40) analyzed WM abnormalities in 153 early-stage drug-naive *de novo* PD and compared these measures with 67 HC from the PPMI database, finding a marginally non-significant interaction between nigral FA and disease status. However, significant interactions between WM regions and disease status have been found in several other non-PPMI studies (32, 38, 106). Analyzing longitudinal PPMI data using VBA, Taylor et al. (66) found significantly increased FA in brainstem, cerebellar, anterior corpus callosal, inferior frontal, and inferior fronto-occipital WM and increased MD in primary sensorimotor and supplementary motor regions. After 1 year, PD patients showed a significantly stronger decline in FA compared to HC in the optic radiation and corpus callosum, as well as parietal, occipital, posterior temporal, posterior thalamic, and vermis GM. The authors postulate that these findings are in line with spatiotemporal patterns of α-synuclein, in both WM and cortical GM. Diffusion tensor imaging–derived metrics of the nigrostriatal tract were shown to have a systematic abnormalities in 50 PD patients from PPMI using tractography; in addition, variations in FA and RD of the nigrostriatal tract were associated with the degree of motor deficits in PD patients (64).

Given the numerous permutations of DTI analysis methods, Mishra et al. (58) performed a systematic comparison of various postprocessing approaches used for identifying WM differences using DTI data from PPMI database. Region-of-interest–based analysis, VBA with varying spatial smoothing, and two widely used skeletonized approaches (TBSS and tensor-based registration with DTI-TK) were compared in a group of 81 early-stage PD and 44 HCs from PPMI. Both skeletonized approaches revealed significant negative correlations for FA with disease duration, although DTI-TK was found to be more accurate for assessing disease progression. However, no analytic techniques showed any group difference in any region between early-stage PD and HC. These types of comparisons provide context for studies that have shown conflicting findings with different analysis pipelines and highlight the importance of standardization of DTI analysis.

To provide a more comprehensive view of early-stage PD-related neuropathological changes, several studies have combined imaging metrics with different underlying pathophysiological correlates. Wei et al. (47) and Pelizzari et al. (41) combined microstructural DTI-derived metrics with perfusion using arterial spin labeling to increase diagnostic accuracy for early-stage PD. In those studies, both FA and cerebral blood flow in the hippocampus, prefrontal cortex, and parietal WM regions were decreased in early-stage PD and mid-late PD compared with HC. In addition, FA was decreased in the SN, while hypoperfusion was observed in the frontal/occipital WM regions. Several authors have also used DTI together with VBM, which can detect subtle brain volumetric changes using structural images, to investigate the relationship between WM tracts, GM volume and PD. These studies found reduced WM microstructural integrity and reduction of GM volume in PD subjects in several regions, such as cingulum, superior longitudinal fasciculus, inferior longitudinal fasciculus, inferior fronto-occipital fasciculus, striatum, and the frontal, temporal, limbic, and paralimbic areas (27, 45, 52, 55, 62, 65, 107).

Sleep disorders, such as rapid eye movement sleep behavior disorder, may coincide with early-stage PD, and the combination of sleep disorders and early-stage PD has been associated with more advanced disease status, despite similar clinical characteristics and cognitive performance (63). Parkinson disease patients with sleep disorders have shown regions of reduced cortical GM volume, as assessed by VBM, and WM changes, most notably reduced FA using TBSS, compared with those who did not have sleep disorders, though not significant when adjusted for multiple comparisons. In a separate study, cortical and subcortical alterations in *de novo* PD patients were observed using VBA-DTI (65); notably, many of these changes occurred in the brainstem, specifically the pontine tegmentum, which has been implicated in the regulation of sleep cycles (108).

Mood disorders, including depression, apathy, and anxiety, have been associated with more advanced PD stages and may be related to dopaminergic depletion (109); however, these neuropsychiatric conditions have also been implicated in early-stage disease and may have a different underlying etiology (62). Gou et al. found no significant WM microstructural differences between depressed and non-depressed PD groups using TBSS (59), a finding that was replicated by Lacey et al. using data from PPMI (53). However, connectivity analysis revealed significant network changes associated with PD patients with depression (59). Parkinson disease with apathy has also been associated with bilateral microstructural alterations in the medial corticostriatal limbic system; more specifically, decreased FA and increased MD were observed in the anterior striatum and pregenual anterior cingulate cortex, along with concomitant serotonergic dysfunction (62).

While most studies have sought to differentiate between PD and HC groups, some studies have assessed the potential of DTI biomarkers to differentiate PD subgroups. In one study, decreased FA was observed in the SN using ROI analysis in early and mid–late PD patients compared with healthy subjects, but there were no significant differences in the same metrics between early-stage PD and mid–late PD groups (47). These results suggest that SN changes occur early in the pathology of PD and rapidly reach a plateau, such that longer disease duration is not indicative of increased nigral microstructural changes. Comparing early-stage PD and neurodegenerative atypical parkinsonism (AP), higher MD in the centrum semiovale, body of the corpus callosum, putamen, external capsule, midbrain, superior cerebellum, and superior cerebellar peduncles has been observed in AP (60). Another study using ROI analysis across PD subgroups found that widespread microstructural changes were present only in late-stage PD groups and not in early and moderate PD groups (61). The authors further suggest that standard DTI methods may not be sensitive to early PD pathology, which may indicate a role for more advanced methods.

### Free-Water Algorithms for DTI Correction

The complex organization of brain tissue, in combination with the relatively large voxel size in dMRI acquisition, results in PVE in diffusion tensor measurements. Consequently, DTI-derived metrics are influenced by the combined contributions of different brain tissue compartments, including CSF and/or extracellular free water (110). Free-water is defined as water molecules that neither experience flow nor are restricted by their surroundings. In the human brain, free water is found in CSF within the ventricles and around the brain parenchyma. For instance, CSF has a relatively large diffusion coefficient compared with that of the brain parenchyma, such that PVE in the periventricular regions and the sulci may overestimate the ADC values by 15–30% (111). Several methods can be employed to remove free-water contributions, such as fluid-attenuated inversion recovery diffusion-weighted imaging (FLAIR-DWI) (112) or the free-water correction algorithm developed by Pasternak et al. (113) for single-shell acquisitions and later extended to multishell acquisitions (114). Planetta et al. (67) used a free-water DTI algorithm in the context of early-stage PD patients taking rasagiline, a monoamine oxidase inhibitor used to treat PD, and found that the +rasagiline group had less free water in the posterior SN and better performance on a coordination task than the –rasagiline group. However, interpretation of changes in free-water measures from single-shell dMRI acquisitions must be interpreted cautiously, as the measures are biased at crossing-fiber regions (115), which make up approximately 90% of WM voxels (116).

### Beyond DTI: Diffusion Kurtosis Imaging

Although DTI is widely used to study WM organization, its inherent assumption of a Gaussian distribution results in an inability to resolve tracts in voxels with complex fiber arrangements (99). To overcome this issue, other techniques such as DKI have been developed. Kurtosis is a statistical measure of the deviation from a Gaussian distribution (which is the assumed distribution for DTI), and thus, DKI-based methods can quantify non-Gaussian diffusion (117). This technique is largely based on the same type of pulse sequences employed for DTI, but DKI requires multishell dMRI at higher *b* values than those conventionally utilized for DTI analysis.

For DKI, the natural logarithm of the diffusion-weighted signal can be approximated by an expansion in terms of the *b* values:

(5) $$\begin{array}{l} ln\left[S\left(b\right)\right]=ln\left[S\left(0\right)\right]-bD_{app}+\frac{1}{6}b^{2}D_{app}^{2}K_{app}+O\left(b^{3}\right) \end{array}$$

where *S*(*b*) is the signal intensity, *D*_*app*_ is the apparent diffusion coefficient, and *K*_*app*_ is the apparent diffusional kurtosis, and the term *O*(*b*^3^) is the Taylor expansion for power of *b* > 2. In the case of Gaussian diffusion, *K*_*app*_ is zero, and Equation (5) reduces to the standard DTI equation. Analogous to DTI, this equation is solvable to yield the diffusion kurtosis tensor $\bar{W}$.

Diffusion kurtosis imaging processing is only slightly more complex than DTI processing, although DKI provides a significantly more complete characterization of water diffusion and tissue structure.

Diffusion kurtosis imaging provides the same set of diffusion parameters as DTI (DKI-FA, DKI-RD, DKI-AD, and DKI-MD), in addition to mean kurtosis (MK), radial kurtosis (RK), and axial kurtosis (AK), which are the most commonly used kurtosis metrics. Similar to DTI, AK is the primary eigenvalue of the apparent kurtosis tensor along the main diffusion direction, whereas RK is the average of the kurtosis coefficients on the equatorial plane. Kurtosis anisotropy is the DKI analog to FA (118).

Even though DKI is more complete than DTI and is able to quantify non-Gaussian diffusion in the brain, DKI-derived diffusion parameters (e.g., DKI-FA) are limited in their sensitivity to detect abnormalities in WM regions with complex fiber arrangements (119). Therefore, the kurtosis indices, such as MK, may be more accurate metrics for WM structural analysis using DKI.

### DKI in Early-Stage PD

Diffusion kurtosis imaging has been used in several studies to assess the early stages of PD. Zhang et al. (82–85) published several studies with DKI in early-stage PD with both cross-sectional and longitudinal designs. In these studies, MK in the SN was significantly increased in PD compared with HCs. Additionally, MK in the SN was positively correlated with the H&Y score staging and part III of the Unified Parkinson's Disease Rating Scale (UPDRS-III). In another study, Surova et al. (86) used DKI to study 105 patients with early-stage PD. They found differences in DTI and DKI metrics between PD subjects and HC in the putamen, thalamus, and superior longitudinal fasciculus, which were also associated with increased disease severity. Guan et al. (42) used ROI analysis of DKI metrics to study advanced and early-stage PD, and MK was found to be significantly lower in bilateral SN in patients with both early-stage and advanced PD than in controls. In addition, MK in the left SN was significantly lower in patients with advanced PD than in those with early-stage PD. However, no differences in FA or MD values were observed between the PD and control groups in that study, and no significant correlations between MK, FA, or MD values and the UPDRS scores were observed.

### Beyond DTI: Neurite Orientation Dispersion and Density Imaging

Neurite orientation dispersion and density imaging is a practical dMRI technique for estimating the microstructural complexity of dendrites and axons *in vivo* (120). Neurite orientation dispersion and density imaging distinguishes between three microstructural environments: intracellular, extracellular, and CSF compartments. Each compartment affects water diffusion in a unique way (121) and gives rise to a separate normalized MR signal. The full normalized signal, *S*(*A*), which includes all environments, is written as follows:

(6) $$\begin{array}{l} S\left(A\right)=\left(1-v_{iso}\right)\cdot \left(v_{ic}A_{ic}+\left(1-v_{ic}\right)A_{ec}\right)+v_{iso}A_{iso} \end{array}$$

where *A*_*ic*_ and *v*_*ic*_ are the normalized signal and volume fraction of the intracellular compartment, respectively; *A*_*ec*_ is the normalized signal of the extracellular compartment; and *A*_*iso*_ and *v*_*ec*_ are the normalized signal and volume fraction of the CSF compartment, respectively (120). By fitting Equation (6), it is possible to obtain *v*_*ic*_, *v*_*iso*_, and the concentration parameter of the Watson distribution (*k*), which is a parameter related to *A*_*ic*_. Using *k*, the orientation dispersion (OD) index can be defined as follows:

(7) $$\begin{array}{l} OD=\frac{2}{\pi }arctan\left(\frac{1}{k}\right) \end{array}$$

Neurite orientation dispersion and density imaging–derived indices have been suggested as imaging biomarkers in early-stage PD, as discussed below. Moreover, NODDI-derived metrics are less sensitive to partial volume effects than DTI (122), which are known to reduce the accuracy of DTI-derived metrics.

### NODDI in Early-Stage PD

Neurite orientation dispersion and density imaging has been used in only two studies in early-stage PD. Andica et al. (87) used both NODDI and tractography to compare *v*_*ic*_, OD, and *v*_*iso*_ between groups of PD and HCs. They found that the contralateral distal *v*_*ic*_ of the nigrostriatal pathway was significantly lower in PD patients than in HCs. However, no correlations were detected between different NODDI indices and disease duration or motor symptom severity. Surova et al. (86) used NODDI, in addition to DKI as discussed above, and ROI analysis in 105 patients with PD, finding increased *v*_*iso*_ in the superior longitudinal fasciculus and decreased *v*_*iso*_ in the corticospinal tract.

### Beyond DTI: Q-Space Diffeomorphic Reconstruction

In addition to limitations of DTI related to non-Gaussian diffusion, other known limitations such as its inability to independently resolve crossing fibers (123) and sensitivity to PVE, result in DTI-derived metrics that reflect a weighted average of multiple diffusion components within a voxel. These limitations reduce the accuracy of DTI-derived metrics and also of WM tractography. For instance, in voxels with multiple tract orientations, a decrease in FA for one of these fiber populations may result in a contradictory increase in the overall FA (124). To partially overcome this issue, dMRI techniques such as QSDR have been developed.

The orientation distribution function (ODF) can be used to characterize the diffusion distribution of fiber populations, thus overcoming crossing fiber limitations. To calculate the ODF, diffusion data can be acquired using a single-shell diffusion sampling scheme, also known as high angular resolution diffusion imaging (HARDI) (123), or a grid sampling scheme, which is known as diffusion spectrum imaging (DSI) acquisition (125). However, studies using ODF to characterize the diffusion distribution may also suffer from partial volume effects. To overcome these effects, the spin distribution function (SDF) can be obtained from generalized Q-sampling imaging (GQI) (126), where SDF represents the proportion of spins undergoing diffusion in different orientations. Q-space diffeomorphic reconstruction is an advanced method to calculate transformed SDFs in any given deformation field that satisfies diffeomorphism (127). Therefore, QSDR can resolve crossing fibers with substantially smaller impact of partial volume effects.

**Figure 6:**
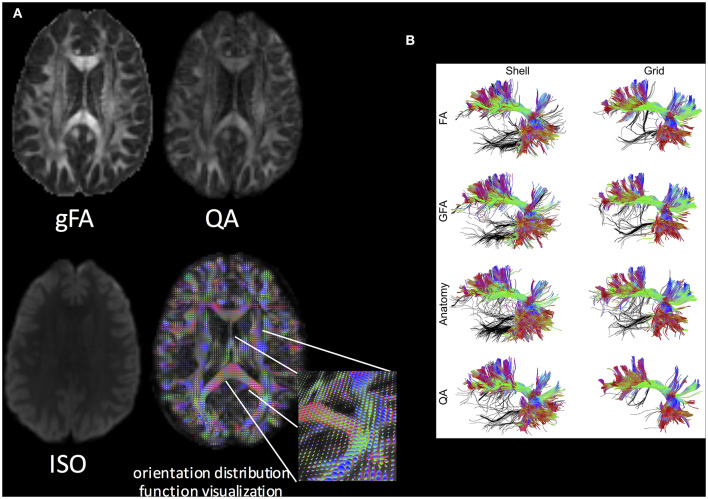
**(A)** Q-space diffeomorphic reconstruction using DSI Studio. gFA, QA, ISO (represents background isotropic diffusion contributed from CSF), and the ODF visualization are shown. **(B)** The performance of the FA-aided, GFA-aided, anatomy-aided, and QA-aided tractographies showing arcuate fasciculus using shell and grid sampling data. The false tracks are colored in black, whereas accurate trajectories are coded by directional color. The tractographies using FA, GFA, and anatomical information show substantially more false tracks than do those using QA. The best performance can be observed in the tractography using QA and the grid dataset. Reproduced with permission from Yeh et al. (128).

The quantitative anisotropy (QA), which is defined as the proportion of spins that undergo diffusion along a given fiber orientation, can characterize the diffusion behavior of a fiber population. Quantitative anisotropy is calculated from the peak orientations on an SDF. Each peak orientation defines a QA value:

(8) $$\begin{array}{l} QA\left(\hat{a}\right)=Z_{0}\left(\psi_{Q}\left(\hat{a}\right)-I\left(\psi_{Q}\right)\right) \end{array}$$

where ψ_*Q*_ is the SDF, *Z*_0_ is the SDF scaling constant, â is the orientation of the fiber defined by the local maximum of the SDF, and *I*(ψ_*Q*_) is the background isotropic component, which can

be approximated by the minimum value of the SDF. With QSDR and the QA index, it is possible to run subsequent analysis, such as VBA (through the normalized QA; nQA = QA/[max QA value]), tractography, and connectome analyses (Figure 6).

### QSDR in Early-Stage PD

All QSDR studies in early-stage PD used patients from the PPMI database. Ghazi Sherbaf et al. (69) published several studies by using this technique utilizing PPMI database. They studied PD subjects with depression and sleep behavior disorder (70–72). Additionally, they found that PD patients exhibited negative correlations between QA and insulinlike growth factor 1 level in several WM locations, including the middle cerebellar peduncle, left and right cingulum, and genu and splenium of the corpus callosum (72); additionally, they demonstrated that the blood marker apolipoprotein A-1 can predict microstructural changes in WM regions in early-stage PD patients with undisturbed cognition and mild motor disability (68). Utilizing QA in a connectome analyses, Haghshomar et al. (74) previously studied the structural correlation of various WM tracts in 81 early-stage PD patients with the whole-blood neutrophil-to-lymphocyte ratio and identified that the QA index correlated with this ratio in the bilateral cingulum, body and left crus of fornix, bilateral corticospinal tract, body and splenium of corpus callosum, and superior cerebellar peduncle. In another study of 85 subjects with PD, connectome analysis of QA revealed a positive association with WM density in bilateral corticospinal tract in the H&Y stage 1 patients, whereas a negative association was observed in the genu of the corpus callosum and bilateral cingulum in H&Y stages 1 and 2 groups (75). In addition, associations between autonomic functional scores and structural brain connectivity in PD were found. Wen et al. (80) used TBSS with graph-theoretical and network-based analyses with QSDR reconstruction to compare WM regional and network features between early-stage PD [tremor-dominant and postural instability and gait difficulty (PIGD) subtypes] and HC groups. Using TBSS and QSDR, tremor-dominant patients showed increased FA and decreased RD and AxD in several areas. Additionally, motor severity had mild to moderate correlations with FA and RD in the genu of the corpus callosum in tremor-dominant subjects, whereas motor severity had strong correlations with FA and RD of multiple association tracts in PIGD subjects. On the other hand, network-based statistical analysis did not reveal any subnetworks with connectivity differences between groups.

Structural network alterations have also been investigated from healthy aging to the prodromal phase of PD to early-stage PD (73). Compared with HCs and *de novo* PD patients, prodromal PD patients showed significantly increased small-world-ness, higher clustering coefficient, and greater local connectivity between regions relating to motor, olfactory, and sleep functions. Parkinson disease patients without hyposmia have shown a significant decrease in global efficiency compared to controls using TBSS with graph-theoretical methods and network-based statistics (81). Additionally, PD patients with hyposmia were shown to exhibit significantly reduced global and local efficiency, as well as a disrupted connection between the right medial orbitofrontal cortex and left rectus, with poorer frontal-related cognitive functioning. Utilizing connectome analysis in 18 early-stage PD and 17 prodromal PD patients, Sanjari Moghaddam et al. (76) investigated the microstructural association of olfaction in prodromal PD as compared to early-stage drug-naive PD patients. Their studies suggested that individuals with prodromal PD have a significantly higher QA as compared to PD patients in bilateral middle cerebellar peduncles and right arcuate fasciculus.

Wen et al. (79) analyzed two different groups of early-stage PD (H&Y stages 1 and 2) using TBSS and QSDR connectome analysis. They found that the earlier stage PD (H&Y stage 1) was associated with higher FA and lower MD and RD in callosal, projection, and association fibers than both HCs and higher-stage PD (H&Y stage 2). The timeline of these alterations between HC and H&Y stage 2 PD was hypothesized to be indicative of compensatory mechanisms arising from early dopaminergic dysfunction in the SN, although further studies are needed to confirm the presence of these neural compensation mechanisms. In addition, motor severity was inversely correlated with FA and positively correlated with MD and RD in PD patients. Moreover, connectome analysis also revealed increased WM density in the aforementioned tracts in PD patients, compared with HCs.

Finally, olfactory dysfunction has been investigated in relation to QSDR findings. Multiple regression analysis in prodromal PD demonstrated positive association between the University of Pennsylvania Smell Identification Test (UPSIT) score and connectivity in left and right subgenual cingulum, right inferior fronto-occipital fasciculus, left corticospinal tract, left parietopontine, left corticothalamic tract, and the body and the splenium of corpus callosum. Sobhani et al. (77) studied olfactory dysfunction in PD, confirming a discriminative role for UPSIT score in identifying WM microstructure changes in early-PD subjects.

### Software for DTI Analysis

Different software packages have been used for diffusion analysis in early-stage PD (Table 2). Most of the available software provides both preprocessing and processing steps within the same pipeline. A standalone preprocessing method, DTIprep, may also be used, which performs an automatic study-specific protocol for DWI/DTI quality control and preparation. After preprocessing, multiple software packages are available to generate DTI-derived metrics. FSL is one of the most widely used software packages for DTI analysis. FDT-FSL employs both linear least squares (LLS) and weighted linear least squares (WLLS) fitting procedures. CAMINO is another open-source software toolkit for dMRI processing. It employs LLS, WLLS, unconstrained non-linear optimization, and the robust estimation of tensors involving the outlier rejection (RESTORE) fitting algorithms. Robust fitting methods (RESTORE and iRESTORE), together with LLS fitting, can also be found in the tolerably obsessive registration and tensor optimization indolent software ensemble (TORTOISE) software. Analysis of Functional NeuroImages (AFNI) is frequently used for fMRI analysis, but it can also be employed for DTI fitting (LLS and non-LLS). Diffusion spectrum imaging Studio is a tractography software tool that maps brain connections and correlates findings with neuropsychological disorders. It works with several dMRI methods, including DTI, GQI, QSDR, dMRI connectome analysis, and generalized deterministic fiber tracking. DTI Studio and Explore DTI are other reliable software packages for DTI fitting and dMRI analysis. FSL's probtrackx (within FDT) and the TRActs Constrained by UnderLying Anatomy (Tracula, within FreeSurfer) are both used for probabilistic tractography; DSI Studio and TrackVis are appropriate for deterministic tractography, whereas MRtrix contains both probabilistic and deterministic algorithms. Matlab toolboxes include the Brain Connectivity Toolbox (BCT) for dMRI-derived structural connectivity analysis, DTI and Fiber Tracking toolbox for DTI fitting and tractography, and NODDI toolbox for NODDI analysis. In several studies, authors used in-house software or MRI workstation software for DTI and DKI analysis.

**Table 2:** List of the main software for dMRI data processing and analysis available.

**Software**	**Diffusion method**	**Link**	**Capabilities**
FMRIB's Diffusion Toolbox (FDT) in FMRIB Software Library (FSL)	DTI	https://fsl.fmrib.ox.ac.uk/fsl/fslwiki	Preprocessing, fitting, and tractography
Tract-Based Spatial Statistics (TBSS) in FSL	DTI	https://fsl.fmrib.ox.ac.uk/fsl/fslwiki	Skeletonized analysis
Camino	DTI, tractography, multifiber and HARDI reconstruction techniques	http://camino.cs.ucl.ac.uk/index.php?n=Main.HomePage	Preanalysis and postanalysis
Tolerably obsessive registration and tensor optimization indolent software ensemble (TORTOISE)	DTI	https://tortoise.nibib.nih.gov/	Prefitting and fitting
Analysis of Functional NeuroImages (AFNI)	DTI	https://afni.nimh.nih.gov/	Fitting
DSI Studio	DTI, GQI, QSDR, DSI, connectometry, tractography	http://dsi-studio.labsolver.org/	Preprocessing and data analysis
DTI Studio	DTI	http://lbam.med.jhmi.edu/	Fitting
Explore DTI	DTI, tractography	http://www.exploredti.com/	DTI MRI and fiber tractography
MRtrix	Tractography	https://www.mrtrix.org/	Fiber tractography analysis
TrackVis	Tractography for DTI/DSI/HARDI/Q-Ball	http://www.trackvis.org/	Fiber tractography analysis
Brain Connectivity Toolbox (BCT)—(MATLAB)	Connectivity	https://sites.google.com/site/bctnet/	Connectivity analysis
DTI and Fiber Tracking (MATLAB)	DTI, tractography	https://www.mathworks.com/matlabcentral/fileexchange/21130-dti-and-fiber-tracking	Fitting and fiber tracking
DTIprep	DWI/DTI quality control and preparation	https://www.nitrc.org/projects/dtiprep	Preprocessing

## Discussion

### Current State of dMRI in Early-Stage PD

Overall, dMRI has been widely used in research settings to investigate WM changes in early-stage PD. In general, DTI acquisition (and similarly DKI) is fairly standardized across vendors and sites, leading to high consistency for data acquisition. The vast majority (56 of 62) of studies included in this systematic review used two *b* values (typically *b* = 1,000 s/mm^2^ and *b* = 0), although three studies used more *b* values (up to four *b* values), and three did not provide this information. More variability is observed in terms of the number of diffusion directions across studies, although the majority used more than 60 directions (37 of 62 studies). Several studies used 30 to 39 directions (seven studies) or 20 to 29 directions (12 studies). Only three studies used fewer than 20 directions. As stated above, more advanced methods require high angular resolution dMRI data, whereas even standard DTI may benefit from improved data acquisition. In general, more than 30 directions can be recommended, and multiple shells may enable improved quantification of dMRI metrics. Despite the similarity in acquisitions, analysis methods can vary widely across studies and are less standardized.

Additionally, the various preprocessing steps for diffusion data can lead to different findings. Echo-planar imaging (EPI) acquisitions suffer from geometric and intensity distortions caused by static magnetic field inhomogeneity, which is worse at higher field strengths; additionally, DTI images are susceptible to the distortions caused by eddy currents induced by large diffusion gradients. There are several methods to correct these issues as part of the preprocessing pipeline. For example, the FSL toolbox includes several tools, including eddy and top-up, which can be used alone or in concert to correct dMRI images. Moreover, the effects of the signal-to-noise ratio can have effect on the accuracy and reproducibility of DTI-derived metrics (129), highlighting the importance of both acquisition and preprocessing steps.

Another important point to consider in light of the dMRI findings in early-stage PD is the effects of the different statistical methods used. Correction of *p*-values can be achieved in multiple ways and may vary across studies. For instance, the threshold-free cluster enhancement is a method for finding significant clusters without having to define binary clusters, whereas cluster-based thresholding methods are used to correct data for multiple comparisons. Often, the data are reported as family-wise error (FWE)-corrected, which means that the family-wise error rate is controlled. It is important to note that correction for multiple comparisons (e.g., Bonferroni or false discovery rate) is compulsory when analysis includes more than two groups (e.g., analysis of variance *post hoc* analysis) and/or when using multiple ROI analysis.

Despite the lack of standardized methods, dMRI methods are increasingly used to understand whether dMRI-derived metrics suggesting WM disorganization are related to clinical presentation. Early-stage PD patients with non-motor symptoms, such as those with olfactory dysfunction or those with substantial SN dopaminergic neuron loss, have been analyzed with various dMRI techniques. Conventional single-tensor DTI models, as well as more sophisticated dMRI models such as DKI and NODDI, have independently suggested diffuse WM changes in early-stage PD, supporting the notion of early axonal damage in PD while simultaneously suggesting that PD pathology may go unrecognized until symptoms appear. Moreover, conventional DTI metrics such as FA and MD have displayed sensitivity to potentially identify earlier symptomatic regions, such as olfactory structures, using correlations with clinical presentations in predetermined ROI analysis. In addition, several studies have considered dMRI changes in GM (10, 45, 65, 66, 86, 107, 122), which may also be implicated in PD pathology. However, as GM lacks the microstructural organization of WM, interpretation of GM-DTI must be approached with caution. In contrast, NODDI has previously been shown to reflect the neurobiology of cortical microstructures (122), suggesting that more advanced dMRI models may enable robust GM characterization in early-stage PD.

However, studies have often displayed heterogeneous results, which may be at least partially attributable to the varying preprocessing and postprocessing steps and statistical approaches utilized. Early-stage PD patients are also heterogeneous in their clinical presentations, and this may further contribute to such varied findings. Although the combination of different dMRI acquisitions and analyses may yield new and more accurate information related to dMRI-derived WM structural disorganization and global connectivity changes in early-stage PD, dMRI has the capability to be developed as a useful neuroimaging tool for diagnosis and prognosis of early-stage PD by rigorously developing and enforcing a set of standardized acquisition and postprocessing tools. This will ultimately provide more reliable dMRI-derived neuroimaging biomarkers that are not only significantly different when compared to HC in early-stage PD, but also attempt to explain their correlations with clinical presentation. Altogether, these will in turn help to understand the neuropathological underpinnings of the progression of PD. Utilizing advanced dMRI methods that overcome some of the known limitations of DTI is also imperative, as measures derived through these advanced dMRI methods may help to understand tract-specific deterioration of WM organization in the progression of PD. Ultimately, ensuring standardization of patient recruitment, data acquisition, postprocessing analytical tools, and statistical approaches could move dMRI toward clinical implementation for identifying dMRI-derived neuroimaging biomarkers.

### Challenges of dMRI in Early-Stage PD

As previously mentioned, conflicting results are often reported in dMRI studies on PD subjects. The small sample size of participants, heterogeneous clinical presentation, and sex imbalance may be responsible for such non-reproducible results reported in the literature. Moreover, the heterogeneity of findings may also be related to the dMRI data acquisition. It is important to emphasize that dMRI acquisitions with fewer than 30 directions may not correctly estimate DTI metrics. For robust estimation of anisotropy, at least 20 unique sampling orientations are necessary, whereas at least 30 unique sampling orientations are required for robust estimation of both tensor orientation and MD. Diffusion schemes with a lower number of sampling orientations may introduce bias and spurious correlations between tensor orientation and apparent diffusion characteristics (130). Hence, acquiring data with at least 45 to 60 diffusion-encoding directions might help to better resolve crossing fibers, may aid in connectome analyses, and can be generally recommended for dMRI data acquisition.

The trend to acquire multishell dMRI data with multiple *b* values is increasing and is also widely recommended. With the advent of simultaneous multislice techniques, it is now clinically feasible to acquire high angular resolution data with multiple shells in clinically acceptable time of approximately 20 min. Acquiring such high angular resolution data with multiple shells permits estimation of advanced dMRI-derived metrics, such as free-water corrected DTI metrics, DKI-derived metrics, and NODDI-metrics. In the future, the sensitivity and specificity of each of these measures should be compared with disease progression to identify the most reproducible, sensitive, and specific dMRI-derived neuroimaging biomarkers for understanding neuropathological underpinnings of PD.

Another possible source of different results lies in the different postprocessing software and statistical approaches used. The algorithms used for tensor fitting of diffusion-weighted data can have substantial effects on the results, not only for PD, but also for other diseases (131, 132). For instance, although LLS fitting model is fast, it incorrectly assumes that data outliers are homogeneously distributed, and therefore it fails to appropriately deweight their contributions. On the other hand, WLLS is slower than the LLS but assigns a weight according to how much the original noise variation is affected by logarithmic transform of the data. While more robust fitting algorithms are available, such as RESTORE and iRESTORE, these are not frequently used because of more complicated pipelines. Identifying the most reproducible statistical approach and the best preprocessing and postprocessing tools are important unmet needs in analysis of dMRI data.

### New Avenues for dMRI in Early-Stage PD

We previously discussed that DTI has several limitations related to an assumption of Gaussian diffusion, presence of crossing fibers, and partial volume effects. Each of the methods presented above that move beyond standard DTI has some aspect that overcomes these limitations, such as DKI to characterize deviations from Gaussian diffusion, NODDI to characterize and minimize partial volume effects, and QSDR to characterize both intravoxel fiber orientation heterogeneity and partial volume effects. Network-based approaches that do not assume the caliber or density of axons in WM, but rather only the orientation of axons, have seen a surge in recent years since PD has been postulated as a network disorder. In several early-stage PD studies, these advanced approaches have been used to overcome DTI-related limitations, although other options are available and have not been investigated. For example, DSI (133) is a technique that can resolve the fiber crossing limitation; however, DSI requires both more time for acquisition than standard DTI and larger pulsed field gradients.

While each of these methods may overcome a fundamental limitation for DTI, these methods may have different limitations themselves (113). For instance, DKI and NODDI suffer from their inherent mathematical assumptions that require data collection methods and analysis that may not be practical or feasible. Interpreting the free-water contribution as applied to DTI is also challenging. Studying network topology through WM-derived structural connectivity may be limited because there is no consensus on the choice of tracking algorithm or edge weights. However, high angular resolution dMRI data acquisition at multiple shells in a clinically acceptable time lends hope in applying and standardizing these advanced dMRI techniques, while simultaneously permitting the incorporation of metrics from these advanced dMRI techniques into network-based analysis.

### Outlook for Future of dMRI in Early-Stage PD

The clinical management of PD faces a significant challenge because moderate to severe neurodegeneration has been shown to be present before the diagnosis is rendered. In addition, the classic presentation of motor disability in PD is shown to co-occur with non-motor symptoms such as changes in mood and behavior, cognitive impairment, sleep disorders, and olfactory dysfunction. The need for neuroimaging biomarkers to detect initial neuropathological changes is crucial to optimize patient care via correct diagnosis and treatment.

Diffusion tensor imaging–based metrics have shown significant but subtle changes in early-stage PD in many areas, such as the motor, premotor, and supplementary motor cortices, corpus callosum, and SN (37, 134, 135). Moreover, non-motor features common to PD, including olfactory dysfunction, REM sleep behavior disorder, cognitive impairment, excessive daytime sleepiness, and depression, can appear in the early stages of PD, providing rationale for screening for PD-like pathology (136). While all of these symptoms have been studied with DTI, the results have been limited or heterogeneous (137, 138). Although hallmark regions such as the SN have been extensively studied using DTI, other regions, along with corresponding hallmarks of PD pathology, should be examined more extensively via advanced dMRI techniques. Additionally, further studies and the inclusion of advanced dMRI methods may aid in establishing more coherent knowledge of WM changes in PD-associated non-motoric symptoms, possibly providing neuroimaging-based biomarkers and thus creating an avenue for advancement of patient care and treatment.

## Data Availability Statement

All datasets generated for this study are included in the article/supplementary material.

## Author Contributions

RW conceived of the presented idea. MB and EK performed the literature search and method review. MB created all figures and tables. MB, EK, VM, and AS wrote the manuscript. All authors edited and approved the manuscript. AS and RW share senior authorship.

## Conflict of Interest

The authors declare that the research was conducted in the absence of any commercial or financial relationships that could be construed as a potential conflict of interest.

## Abbreviations

dMRI: Diffusion MRI
PD: Parkinson disease
SN: substantia nigra
MRI: Magnetic resonance imaging
VBM: voxel-based morphometry
fMRI: functional MRI
rs-fMRI: Resting-state-fMRI
HC: healthy controls
DTI: Diffusion tensor imaging
WM: white matter
FA: fractional anisotropy
MD: mean diffusivity
AxD: axial diffusivity
RD: radial diffusivity
DKI: diffusion kurtosis imaging
H&Y: Hoehn & Yahr
PPMI: Parkinson's Progression Markers Initiative
NODDI: neurite orientation dispersion and density imaging
QSDR: Q-space diffeomorphic reconstruction
ROI: region of interest
VBA: voxel-based analysis
TBSS: Tract-Based Spatial Statistics
CSF: cerebrospinal fluid
ASL: arterial spin labeling
CBF: cerebral blood flow
GM: gray matter
AP: atypical parkinsonism
MK: mean kurtosis
RK: radial kurtosis
AK: axial kurtosis
UPDRS-III: Unified Parkinson's Disease Rating Scale
ODF: orientation distribution function
HARDI: high angular resolution diffusion imaging
DSI: diffusion spectrum imaging
SDF: spin distribution function
GQI: generalized Q-sampling imaging
QA: quantitative anisotropy
PIGD: postural instability and gait difficulty
NBS: network-based statistics
LLS: linear least squares
WLLS: weighted linear least squares
RESTORE: robust estimation of tensors involving the outlier rejection
TORTOISE: tolerably obsessive registration and tensor optimization indolent software ensemble
AFNI: Analysis of Functional NeuroImages
Tracula: TRActs Constrained by UnderLying Anatomy
BCT: Brain Connectivity Toolbox.
